# Effects of puerarin on the pharmacokinetics of astragaloside IV in rats and its potential mechanism

**DOI:** 10.1080/13880209.2020.1746362

**Published:** 2020-05-01

**Authors:** Huan Zhang, Jiaying Song, Huizhen Dai, Yanchao Liu, Lili Wang

**Affiliations:** a Department of Ophthalmology, Affiliated Hospital of Weifang Medical University, Weifang, China; b Department of Hematology, Affiliated Hospital of Weifang Medical University, Weifang, China; c Department of Emergency, Affiliated Hospital of Weifang Medical University, Weifang, China; d Operating Room, Affiliated Hospital of Weifang Medical University, Weifang, China

**Keywords:** Drug–drug interaction, CYP3A4, P-gp, metabolism, transport

## Abstract

**Context:**

Puerarin and astragaloside IV (AS-IV) are sometimes used together for the treatment of disease in Chinese clinics, however, the drug–drug interaction between puerarin and AS-IV is still unknown.

**Objective:**

This study investigates the effects of puerarin on the pharmacokinetics of astragaloside IV in rats and clarifies its main mechanism.

**Materials and methods:**

The pharmacokinetic profiles of oral administration of astragaloside IV (20 mg/kg) in Sprague-Dawley rats, with or without pre-treatment of puerarin (100 mg/kg/day for 7 days) were investigated. The effects of puerarin on the transport and metabolic stability of AS-IV were also investigated using Caco-2 cell transwell model and rat liver microsomes.

**Results:**

The results showed that puerarin could significantly increase the peak plasma concentration (from 48.58 ± 7.26 to 72.71 ± 0.62 ng/mL), and decrease the oral clearance (from 47.5 ± 8.91 to 27.15 ± 9.27 L/h/kg) of AS-IV. The Caco-2 cell transwell experiments indicated that puerarin could decrease the efflux ratio of astragaloside IV from 1.89 to 1.26, and the intrinsic clearance rate of astragaloside IV was decreased by the pre-treatment with puerarin (34.8 ± 2.9 *vs*. 41.5 ± 3.8 μL/min/mg protein).

**Discussion and conclusions:**

These results indicated that puerarin could significantly change the pharmacokinetic profiles of astragaloside IV, via increasing the absorption of astragaloside IV or inhibiting the metabolism of astragaloside IV in rats.

## Introduction

Astragaloside-IV (AS-IV) is a high-purity drug extracted from *Radix Astragali*, which is always used as the primary tonic herbs in China (Deng et al. 2016; Zhao et al. 2017). AS-IV has been reported to have pharmacological effects of hepatoprotective, antiviral, anti-inflammatory, antinociceptive, antihypertensive and immunostimulant activities (Zhang et al. 1992; Bedir et al. 2000; Yang et al. 2001; Li and Cao 2002). However, the oral bioavailability of AS-IV is very low (Gu et al. 2004). The low oral bioavailability may be contributed to many factors, including physicochemical factors (i.e., solubility and dissolution), physiological factors (i.e., intestinal absorption, efflux and first-pass metabolism) as well as the pharmacokinetic factors.

Puerarin is a kind of isoflavone isolated from Chinese medicine *Pueraria*, which is widely known as Gegen (Chinese name) in traditional Chinese medicine. Puerarin is also supplied as a food source, medicine, fodder, and it frequently applied to treat fever, liver injury, weight loss, and toxicities (Wong et al. 2011; Zhou et al. 2014). Its extract puerarin is always used in the treatment of cardiovascular diseases and diabetes, due to its wide spectrum of pharmacological properties such as vasodilation, cardioprotection, neuroprotection, antioxidant and anticancer (Zhou et al. 2014). Meanwhile, drug–drug interaction is also important. In previous studies, puerarin has been reported had the inhibitory effects on the CYP 450 enzymes, such as CYP3A4, CYP2B6, CYP2C9, and *P-gp*, which are related to the metabolism and absorption of the drug (Zheng et al. 2010; Guo et al. 2014; Kim et al. 2014; Liu et al. 2015).

Co-administration of different herbs is very common in traditional Chinese medicine. For example, some Chinese patent drugs for the treatment of cervical spondylosis and its complications were mixed of several kinds of herbs including puerarin and AS-IV. Drug-drug interaction should also be considered, since this could strongly affect the pharmacodynamics and pharmacokinetic of co-administrated drugs. A previous study has investigated the effect of AS-IV on the pharmacokinetics of puerarin in rats, and the results indicated that AS-IV decreased the systemic exposure of puerarin (Liu et al. 2019). However, whether puerarin affects the pharmacokinetics of AS-IV is still unknown. To the best of our knowledge, there are little data available for the effect of puerarin on the pharmacokinetics of AS-IV and its potential mechanism.

In this study, the effect of puerarin on the pharmacokinetics of AS-IV was studied. The *in vivo* pharmacokinetics of AS-IV in rats with or without puerarin pre-treatment were determined. Additionally, the effects of puerarin on the metabolism stability of AS-IV were investigated with rat liver microsomes and the Caco-2 cell transwell model.

## Materials and methods

### Chemicals

Puerarin (purity >98%) and AS-IV (purity >98%) was obtained from shanghai Standard Biotechnology Co., Ltd (Shanghai, China). Acetonitrile and methanol were purchased from Fisher Scientific (Fair Lawn, NJ, USA). Dulbecco’s modified Eagle’s medium (DMEM) and non-essential amino acid (NEAA) solution were purchased from Thermo Scientific Corp. (Logan, UT, USA). Foetal bovine serum (FBS) was obtained from GIBCO BRL (Grand Island, NY, USA). Penicillin G (10,000 U/mL) and streptomycin (10 mg/mL) were purchased from Amresco (Solon, OH, USA). Hanks’ balanced salt solution (HBSS) was purchased from GIBCO (Grand Island, NY, USA). Ultrapure water was prepared with a Milli-Q water purification system (Millipore, Billerica, MA, USA). All other chemicals were of analytical grade or better.

### Animal experiments

Male Sprague-Dawley rats weighing 230–250 g were provided by Shanghai SLAC Laboratory Animal Co., Ltd (Shanghai, China). Rats were bred in a breeding room at 25 °C with 60 ± 5% humidity and a 12-h dark/light cycle. Tap water and normal chow were given *ad libitum*. All of the experimental animals were housed under the above conditions, for a three-day acclimation period and fasted overnight before the experiments. All experimental procedures and protocols were reviewed and approved by the Animal Care and Use Committee of Affiliated Hospital of Weifang Medical University and were in accordance with the National Institutes of Health guidelines regarding the principles of animal care.

### *In vivo* pharmacokinetic study

To evaluate the effects of puerarin on the pharmacokinetics of AS-IV, the rats were divided into two groups of six animals each. The test group was pre-treated with puerarin at a dose of 100 mg/kg/day (dissolved directly in normal saline containing 0.5% methylcellulose at a concentration of 2 mg/mL) for 7 days before the administration of AS-IV. Next, AS-IV was orally administered to rats by gavage at a dose of 20 mg/kg (Du et al. 2005; Song, Li, et al. 2014; Song, Zheng, et al. 2014; Wang et al. 2019). Blood samples (250 μL) were collected into heparinized tubes via the *oculi chorioideae* vein at 0.083, 0.33, 0.5, 1, 2, 4, 6, 8, 10, 12 and 24 h after the oral administration of puerarin. The blood samples were centrifuged at 3500 rpm for 5 min. The plasma samples that were obtained were stored at −40 °C until analysis.

### LC-MS/MS determination of as-IV

The determination of warfarin was performed on Agilent 1290 series liquid chromatography system and an Agilent 6470 triple-quadruple mass spectrometer (Palo Alto, CA, USA). The HPLC/MS conditions and sample preparation were basically according to a validated HPLC method described elsewhere (Zhang et al. 2019). The chromatographic analysis of puerarin was performed on a Waters X-Bridge C18 column (3.0 × 100 mm, i.d.; 3.5 μm, USA) at room temperature (25 °C). The mobile phase was water (containing 0.1% formic acid) and acetonitrile (30:70, v: v) with isocratic elution at a flow rate of 0.2 mL/min, and the analysis time was 4 min.

The mass scan mode was positive MRM mode. The precursor ion and product ion are m/z 807.1→627.2 for AS-IV, and m/z 321.4→207.1 for IS. The collision energy for AS-IV and IS were 30 and 20 ev, respectively. The MS/MS conditions were optimised as follows: fragmentor, 110 V; capillary voltage, 3.5 kV; Nozzle voltage, 500 V; nebuliser gas pressure (N_2_), 40 psig; drying gas flow (N_2_), 10 L/min; gas temperature, 350 °C; sheath gas temperature, 400 °C; sheath gas flow, 11 L/min.

### Cell culture

The Caco-2 cell line was obtained from the American Type Culture Collection (Manassas, VA, USA), and it was performed according to the previous study. The Caco-2 cells were cultured in DMEM high glucose medium containing 15% FBS, 1% NEAA and 100 U/mL penicillin and streptomycin. The cells were cultured at 37 °C with 5% CO_2_. For transport studies, the cells at passage 40 were seeded on transwell polycarbonate insert filters (1.12 cm^2^ surface, 0.4 μm pore size, 12 mm diameter; Corning Co-star Corporation, MA, USA) in 12-well plates at a density of 1 × 105 cells/cm^2^. Cells were allowed to grow for 21 days. For the first seven days, the medium was replaced every two days, and then daily. The transepithelial electrical resistance (TEER) of the monolayer cells was measured using Millicell ERS-2 (Millipore Corporation, Billerica, MA, USA), and TEER exceeding 400 Ω·cm^2^ was used for the flux experiment. The integrity of the Caco-2 monolayers was confirmed by the paracellular flux of Lucifer yellow, which was less than 1% per hour. The alkaline phosphatase activity was validated using an Alkaline Phosphatase Assay Kit. The qualified monolayers were used for transport studies.

### Effects of puerarin on the absorption of as-IV in the Caco-2 cell transwell model

Before the transport experiments, the cell monolayers were rinsed twice using warm (37 °C) Hanks’ balanced salt solution (HBSS), then the cells were incubated at 37 °C for 20 min. After preincubation, the cell monolayers were incubated with AS-IV in fresh incubation medium added on either the apical or basolateral side for the indicated times at 37 °C. The volume of incubation medium on the apical and basolateral sides was 0.5 mL and 1.5 mL, respectively, and a 100 μL aliquot of the incubation solution was withdrawn at the indicated time points from the receiver compartment and replaced with the same volume of fresh pre-warmed HBSS buffer. The inhibitory effects of *P-gp* inhibitors on the AS-IV flux by Caco-2 cells were investigated by adding 50 μM puerarin to both sides of the cell monolayers and pre-incubating the sample at 37 °C for 30 min. The permeability of AS-IV (2 μM) in all of the above condition s for both directions, i.e., from the apical (AP) side to the basolateral (BL) side and from the BL side to the AP side, was measured after incubation for 30, 60, 90 and 120 min at 37 °C. In addition, the efflux activity of *P-gp* was validated using a typical *P-gp* substrate digoxin (25 μM).

The apparent permeability coefficient (*P_app_*) was calculated using the equation of Artursson and Karlsson:

$$P_{\mathrm{app}}=\left(\Delta Q/\Delta t\right)\times \left[1/\left(A\times C_{0}\right)\right]$$

where *P_app_* is the apparent permeability coefficient (cm/s), ΔQ/Δt (μmol/s) is the rate at which the compound appears in the receiver chamber, *C_0_* (μmol/L) is the initial concentration of the compound in the donor chamber and A (cm^2^) represents the surface area of the cell monolayer. Data were collected from three separate experiments, and each was performed in triplicate.

### Effects of puerarin on the metabolic stability of as-IV in rat liver microsomes

Rat liver microsomes were used to investigate the effects of puerarin on the metabolism clearance of AS-IV, and the assay conditions and reaction mixtures were similar to those reported previously (Wang et al. 2017; Yan et al. 2017). In brief, 30 μL rat liver microsome (20 mg/mL), 12 μL AS-IV solution (100 μM) and 1113 μL PBS buffer (0.1 M, *pH* 7.4) were added to the centrifuge tubes on ice. There was a 5 min preincubation step at 37 °C before initiating the reaction by adding NADPH-generating system (45 μL) into the microsomal suspension. The effects of puerarin or ketoconazole (a positive CYP3A4 inhibitor) on the metabolic stability of AS-IV were investigated by adding 10 μM of puerarin or ketoconazole (12 μL, final concentration of 0.1 μM) to rat liver microsomes and pre-incubating them for 30 min at 37 °C, followed by the addition of NADPH-generating system. Aliquots of 100 μL were collected from the reaction volumes at 0.083, 0.167, 0.33, 0.5, 1, 2, 4, 8, 12 and 24 h after the addition of AS-IV, and 200 μL ice-cold acetonitrile containing esculin was added to terminate the reaction. All the experiments were performed in triplicate. The subsequent sample preparation method was the same as the plasma sample preparation method, and the concentration of AS-IV was determined by LC-MS.

The *in vitro* half-life (*t*_1/2_) was obtained using the equation: *t*_1/2_ = 0.693/*k*; V (μL/mg) = volume of incubation (μL)/protein in the incubation (mg); Intrinsic clearance (Clint) (μL/min/mg protein) = V × 0.693/*t*_1/2_.

### Data analysis

The pharmacokinetic parameters, including the area under the plasma concentration-time curve (*AUC*), maximal plasma concentration (*C_max_*), the time for the maximal plasma concentration (*T_max_*), and the mean residence time (*MRT*) were calculated using the DAS 3.0 pharmacokinetic software (Chinese Pharmacological Association, Anhui, China).

The differences between the mean values were analysed for significance using a one-way analysis of variance (ANOVA). Values of *p <* 0.05 were considered to be statistically significant.

## Results

### Effects of puerarin on the pharmacokinetics of as-IV

**Figure 1.**
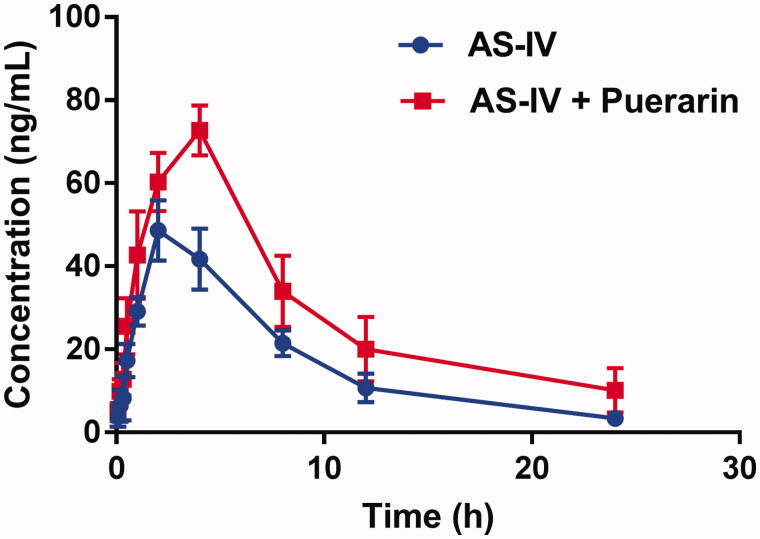
The pharmacokinetic profiles of AS-IV in rats (six rats in each group) after the oral administration of 20 mg/kg AS-IV with or without puerarin pre-treatment (100 mg/kg/day for 7 days). Each point represents the average ± S.D. of six determinations.

The mean plasma concentration-time curves of AS-IV with or without puerarin are shown in Figure 1, and the pharmacokinetic parameters were calculated using the noncompartmental method with the DAS 3.0 pharmacokinetic software (Chinese Pharmacological Association, Anhui, China). The pharmacokinetic parameters are summarised in Table 1.

**Table 1.** Pharmacokinetic parameter of AS-IV in rats after intragastrical administration of AS-IV (20 mg/kg; *n* = 6, Mean ± S.D.) with or without treatment of puerarin.

Parameter	Control	Pre-treatment of Puerarin
*T*_max_ (h)	2.0 ± 0.22	3.98 ± 0.15*
*C*_max_ (ng/mL)	48.58 ± 7.26	72.71 ± 6.02*
*t*_1/2_ (h)	4.47 ± 0.39	6.48 ± 4.80*
AUC _(0-t)_ (μg·h/L)	419.67 ± 84.03	709.95 ± 164.85*
MRT (h)	6.80 ± 0.38	7.71 ± 7.83*
CLz/F(L/h/kg)	47.50 ± 8.91	27.15 ± 9.27*

*p < 0.05 indicate significant differences from the control.

As shown in Figure 1, the peak plasma concentration (*C_max_*) of AS-IV increased from 48.58 ± 7.26 to 72.71 ± 0.62 ng/mL (*p* < 0.05), after the pre-treatment with puerarin, the difference was significant (*p* < 0.05). And the AUC_0-t_ also significantly increased from 419.67 ± 19.67 s to 709.95 ± 164.85 μg·h·L^−1^ (*p* < 0.05). Both results suggested that the administration of puerarin could improve the concentration of AS-IV in the blood plasma. At the same time, puerarin prolonged the half-life (*t*_1/2_) and the mean residence time (*MRT*) of AS-IV, declined clearance rate. These results indicated the metabolism of AS-IV was inhibited which may lead to the plasma concentration of AS-IV increased.

### Effects of puerarin on the bidirectional transport of as-IV across Caco-2 cells

**Figure 2.**
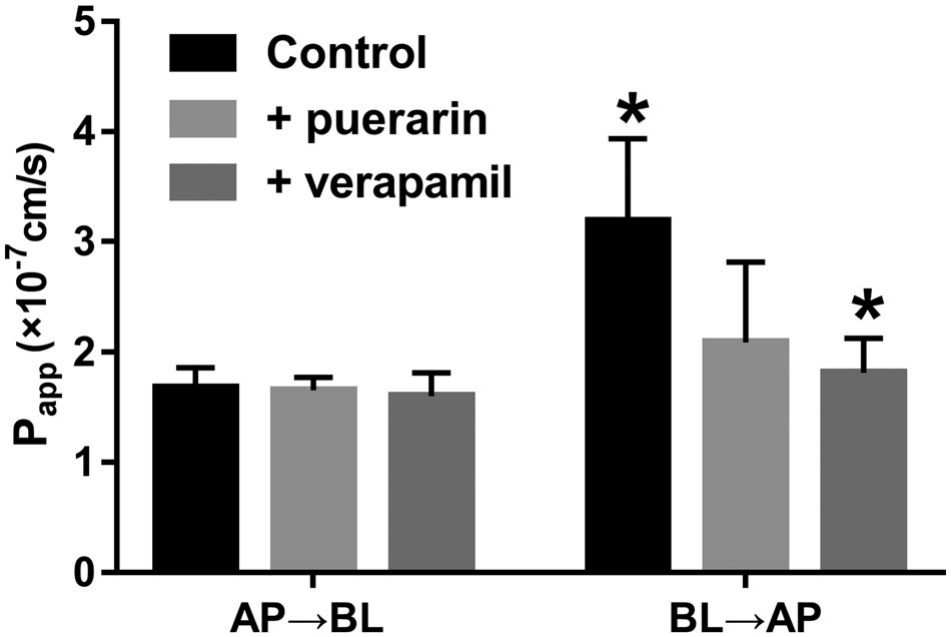
Effects of puerarin or verapamil on the transport of AS-IV from the apical to basolateral side or the opposite direction, Caco-2 cell monolayers were incubated at 37 °C in HBSS (pH 7.4), and AS-IV (2 µM) were added to the apical or basolateral side, verapamil or puerarin were also added to the donor chamber with AS-IV. *Significant differences (*p* < 0.05) were seen compared to the control sample. Each point represents the mean ± SD of three determinations.

The Caco-2 cells *in vitro* model were employed to study the effects of puerarin on the transport of AS-IV. In order to validate the efflux activity of *P-gp*, digoxin a typical *P-gp* substrate and verapamil a typical *P-gp* inhibitor was used. The results indicated that the efflux ratio of digoxin was 9.88 and it was suspended when verapamil was present. The results indicated that the efflux activity of *P-gp* was qualified for the experiment. As shown in Figure 2, the *P_appAB_* and *P_appBA_* of AS-IV were 1.68 ± 0.18 × 10^−7^ and 3.18 ± 0.75 × 10^−7 ^cm/s, respectively. The *P_appBA_* was much higher than the *P_appAB_* of AS-IV, and the efflux ratio was 1.89. These results indicated that there might be efflux transporters related to the transport of AS-IV. In the presence of puerarin, the *P_appBA_* significantly decreased from 1.68 ± 0.18 × 10^−7^ to 2.09 ± 0.72 × 10^−7^. Whereas, the *P_app_* values from AP side to BL side decreased by the treatment of puerarin (3.18 ± 0.75 × 10^−7^ *vs.* 2.63 ± 0.52 × 10^−7^). The efflux ratio was decreased from 1.89 to 1.26, which suggested the efflux of AS-IV was inhibited after the administration of puerarin. Similarly, the efflux ratio decreased from 1.89 to 1.13 in the presence of verapamil, and the efflux of AS-IV was also inhibited. These results revealed that puerarin could accelerate the absorption of AS-IV via inhibiting the activity of *P-gp*.

### Effects of puerarin on the metabolic stability of as-IV in rat liver microsomes

Through rat liver microsomes, the effect of puerarin on the metabolic stability of AS-IV can be investigated. The metabolic half-life (*t*_1/2_) of AS-IV was 33.4 ± 4.1 min, while the presence of puerarin prolonged the *t*_1/2_ of AS-IV to 39.8 ± 5.3 min, and the difference was significant (*p* < 0.05). Meanwhile, puerarin decreased the intrinsic clearance rate of AS-IV from 41.5 ± 3.8 to 34.8 ± 2.9 μL/min/mg protein. These results demonstrated that puerarin inhibited the metabolism of AS-IV in rat liver microsomes and decreased the intrinsic clearance rate in rat liver microsomes. This was consistent with the results of pharmacokinetics study.

## Discussion

The co-administration of different herbs is commonly accepted in traditional Chinese medicine. And the drug-drug interaction plays a vital role during the co-administration. AS-IV is poorly absorbed and has low bioavailability and it is susceptible to external factors. Previous studies paid little attention to the effect of other herbs on the pharmacokinetics of AS-IV. The subject of this study was to explore the pharmacokinetics of AS-IV when co-administrated with puerarin.

In the pharmacokinetics study, the results indicated that puerarin inhibited the metabolism of AS-IV, increased the plasma concentration of AS-IV, where the *C_max_* and *AUC_(0-t)_* increased significantly, the half-life (*t*_1/2_) prolonged and clearance rate decreased. To investigate the effect of puerarin on the metabolism stability of AS-IV *in vitro*, the rat liver microsomes was adopted. Moreover, the results suggested that puerarin inhibited the metabolism of AS-IV in rat liver microsomes, which verified the results of pharmacokinetics study.

Via the Caco-2 cell monolayer model, we studied the effect of puerarin on the absorption of AS-IV and expounded its potential mechanism. The results showed that the *P_app_* values from the BL side to the AP side decreased, and with verapamil as reference, we concluded that puerarin inhibited the activity of *P-gp*, decreased the efflux of AS-IV. As previous studies reported, puerarin inhibits the activity of CYP3A4 and *P-gp* (Guo et al. 2014; Kim et al. 2014), which are closely related to the metabolism and transport of AS-IV. Thus, we surmised that puerarin might increase the absorption of AS-IV by inhibiting *P-gp* mediated its efflux or inhibiting CYP3A4 mediated metabolism.

## Conclusions

Puerarin significantly influences the pharmacokinetics of AS-IV, when they are co-administered. According to the *in vivo* pharmacokinetic study and rat liver microsome incubation systems, we inferred that puerarin could increase the system exposure of AS-IV through inhibiting the activity of CYP3A4, which was related to the metabolism of AS-IV. In addition, the result of Caco-2 cell monolayer model illustrated puerarin mediated efflux of AS-IV via inhibiting the activity of *P-gp*. Therefore, the drug-drug interaction between puerarin and AS-IV is a considerable factor when they are co-administered. However, this research is a mechanistic study, the potential interaction between puerarin and AS-IV need to be assessed by some *in vivo* investigations in some conditions close to human.
